# Novel Strategies to Reach and Engage Older Veterans During COVID-19

**DOI:** 10.1093/geroni/igab046.785

**Published:** 2021-12-17

**Authors:** Amanda Peeples, Kim Van Orden

## Abstract

The COVID-19 pandemic and associated public health measures to prevent its spread have important implications for the health and wellbeing of older Veterans. Prior to the pandemic, social isolation was already recognized as a risk for older adults, contributing to increased risk of depression, physical inactivity, and mortality. Stay-at-home orders, social distancing, and transitions to new ways of delivering care have meant that many of the ways in which older Veterans connect with VA and others have changed. Older Veterans and Veterans with serious mental illness (SMI) are especially vulnerable to experience negative impacts from social isolation and loneliness. This symposium will present on four novel and adapted strategies for engaging with older Veterans during the COVID-19 pandemic and beyond: 1) VA Connection Plans, a whole health intervention to promote social connections for older Veterans with and without SMI (Peeples); 2) telehealth adaptations to PEER, an in-person, peer-delivered exercise intervention for older Veterans with SMI (Muralidharan); 3) VA Compassionate Contact Corps, a VA Voluntary Service program to connect older Veterans with friendly volunteers via telephone (Sullivan); and 4) group telehealth interventions to foster social connection among older Veterans and their families (Weiskittle). Kim Van Orden, geropsychologist and director of the Hope Lab (Helping Older People Engage) at the University of Rochester Medical Center, will serve as discussant.

